# Validity of using perceived exertion to assess muscle fatigue during back squat exercise

**DOI:** 10.1186/s13102-023-00620-8

**Published:** 2023-02-04

**Authors:** Hanye Zhao, Dasom Seo, Junichi Okada

**Affiliations:** 1 Graduate School of Sport Sciences, Waseda University, Tokorozawa, Saitama Japan; 2 Faculty of Sport Sciences, Waseda University, Tokorozawa, Saitama Japan; 3 Graduate School of Sport Sciences, Waseda University, Mikajima 2-579-15, Tokorozawa, Saitama 359-1192 Japan

**Keywords:** Perceived exertion, Neuromuscular fatigue, Borg scale, Surface electromyography, Resistance training

## Abstract

The rating of perceived exertion (RPE) scale has been found to reflect physiological responses, and this study aimed to assess the validity of using the Borg CR-10 scale and velocity loss to evaluate muscle fatigue quantified by surface electromyography during back squat (BS) exercise. A total of 15 collegiate male athletes underwent three non-explosive BS tasks comprising low, medium, and high volumes at 65% of their one-repetition maximum. RPEs, spectral fatigue index (SFI), and velocity loss during BS exercise were assessed throughout the trials. Significant differences in overall RPE (*p* < 0.001) and average SFI (*p* < 0.05) were observed between the conditions, whereas no significant difference was observed in average velocity loss. Significant increases in RPE and SFI (*p* < 0.001) were observed within the exercise process, whereas a significant increase in velocity loss was not observed. Correlation analyses indicated a significant correlation between RPE and SFI obtained during exercise (*r* = 0.573, *p* < 0.001). However, no significant correlation was observed between velocity loss and SFI. These results demonstrated that RPE could be used as a muscle fatigue predictor in BS exercise, but that velocity loss may not reflect muscle fatigue correctly when participants cannot and/or are not required to perform BS explosively. Furthermore, practitioners should not use velocity loss as a muscle fatigue indicator in some resistance exercise situations, such as rehabilitation, beginner, and hypertrophy programs.

## Background

Muscle fatigue is a common phenomenon in everyday life. It can be perceived as subjective feelings such as fatigue and tiredness. Muscle fatigue associated with many internal and external stress and stimulus [1]. When performing resistance training, muscle fatigue is thought to be an inevitable phenomenon. Further, because of accompanying impairments in force and/or power-generating capacity, muscle fatigue is related to decrease in exercise performance such as peak velocity and/or power output, [2]. Furthermore, muscle fatigue also is associated with acute injury risks and some chronic soreness [3, 4]. Muscle fatigue can be manifested by many physiological measures (e.g., blood lactate concentration and muscle force output) [2, 5]. Surface electromyography (sEMG) is a non-invasive neuromuscular function assessing device and has been widely applicated in medical, neuroscience and sports science areas [6, 7]. Fatigue-induced neuromuscular responses can be estimated using electromyographic signals during muscle contractions [7, 8]. In the past decades, ﻿sEMG have been shown to give trusty information regarding the underlying mechanism of neuromuscular fatigue [9]. When using sEMG as muscle fatigue indicator, physiological-related power spectral changes (e.g., muscle fiber conduction velocity) determine the representation of muscle fatigue [10]. The variation in the power spectral characteristic causes a shift in power spectrum toward lower frequencies. Accordingly, mean and median frequencies of power spectrum are commonly thought as muscle fatigue estimators [7].

It is very important for professionals in this field, such as personal trainers, coaches, and physical therapists, to grasp the fatigue conditions of those with whom they work [2]. Because of the widespread application of sEMG-based muscle fatigue assessments in sports science areas, some practitioners have started using sEMG in resistance exercise situations. However, because of limitations, such as the expensive price and analysis techniques, it is not realistic for most practitioners to widely use sEMG-based muscle fatigue assessments during resistance exercise situations [7]. Even if practitioners can afford relatively cheap sEMG devices, they usually have very limited reliability or are incapable of muscle fatigue assessment. As for experimental sEMG devices with acceptable reliability and functions, they are much more expensive and almost unaffordable for individual trainers and coaches. Moreover, real-time feedback on muscle fatigue is required in many exercise scenarios (e.g., rehabilitation), which cannot be achieved by monitoring spectral changes because they occur over a much longer time frame [11].

Physiological responses are reliable variables for monitoring exercise-induced metabolic changes and, therefore, reflect muscle fatigue responses during exercises [2]. For example, it has been suggested that blood lactate concentration can be viewed as an important indirect muscle fatigue marker [1, 12, 13]. However, blood lactate measurements are often accompanied by inevitable invasive techniques (e.g., fingertip puncture) that can cause discomfort in participants. Furthermore, blood lactate can easily be affected by many factors. For example, blood lactate also changes significantly along with relative intensity and an array of resistance exercises [14, 15]. In this situation, it is difficult for practitioners to separate blood lactate changes that are only induced by muscle fatigue. Accordingly, these measures may be unsuitable for exercise situations. Recently, velocity-based training has become very popular in resistance exercise scenarios because of its positive impact on athletic performance and because a wide range of tools are currently available for velocity monitoring [16, 17]. These devices usually measure exercise velocity through rotary encoders, accelerometers, and linear position transducers with a cable attached to weights. The displacement changes of the weights over time during lifting are recorded for velocity calculation. Within the popularization of velocity measures, velocity loss has been recommended as a new fatigue indicator for resistance exercises [2]. For example, it has been reported that velocity loss is correlated significantly with metabolic and mechanical measurements of fatigue during explosive back squat (BS) exercise [2]. However, most studies on velocity loss have examined only the validity of velocity loss when the exercise is performed explosively [2, 18]. Thus, velocity loss might be inappropriate for assessing muscle fatigue in many other resistance exercise programs. For example, for muscular hypertrophy-aimed resistance training programs, the amount of time of a certain load on muscle is crucial for muscle protein synthesis [19]. Moreover, it might be inappropriate for some people to perform explosive exercises (e.g., novice lifters and people with injuries). These people are usually lack of proper technique and joint stability [20]. Accordingly, the validity of velocity-based muscle fatigue assessment might be inappropriate or inadequate when peak velocity and power are no longer the target of exercise program.

The Borg rating of perceived exertion (RPE) scale is a simple and reliable exercise intensity estimator that combines numbers and verbal [21, 22]. RPE has been reported to reflect physiological changes such as heart rate during aerobic exercise [22, 23]. Over the last two decades, RPE has been widely applicated for prescription of intensity of resistance exercise programs. For example, RPE was reported to associate with the percentage of one-repetition maximum strength (1RM) of selected resistance exercises [14]. ﻿More recently, resistance exercise-specific and aerobic exercise-specific RPE scales also have been devised that can be used to prescribe various of exercises [24]. Despite style differences between scales, RPE is an easy-to-use and effective measure for assessing resistance exercise intensity [25]. Consequently, it may also be used as muscle fatigue estimator in resistance exercise situations. However, because the sEMG signal would become unstable during dynamic muscle contractions (such as resistance exercise performed with dynamic contractions), quantifications of muscle fatigue in these exercise situations require considerable technique and competence [7]. Accordingly, the relationship between RPE and muscle fatigue has only been inspected for certain isometric exercises [26, 27].

In recent years, mathematical simulation-based methods to assess muscle fatigue using sEMG signals have been developed [8, 11]. Thus, a relationship between RPE and muscle fatigue in multi-joint dynamic resistance exercises might be established by using the new sEMG processing technique. Our previous study has indicated the possibility of using RPE to predict muscle fatigue assessed by the new mathematical simulation-based method using sEMG during single-joint resistance exercises [28]. If the relationship could be established, the use of RPE-based fatigue assessment would expand to multi-joint resistance exercises.

The interdependence between physiological and perceptual responses during exercises indicates that RPE may be a valid method for assessing muscle fatigue during resistance exercises. However, velocity loss might be inappropriate for muscle fatigue assessment when resistance exercise is no longer performed explosively. Accordingly, this study’s aims are as follows: (1) to examine the validity of using RPE to assess muscle fatigue quantified by new sEMG techniques, and (2) to compare the validity of using velocity loss as a fatigue indicator during non-explosive BS exercise. We hypothesized that (1) RPE and muscle fatigue would change similarly, and a significant correlation between muscle fatigue and RPE could be observed; however, (2) velocity loss may not reflect muscle fatigue correctly when BS exercise is no longer performed explosively.

## Methods

### Experimental designs

﻿ This study was designed to verify the validity of using RPE scores to predict muscle fatigue during BS exercise and to compare this with the use of velocity loss as a muscle fatigue indicator. BS were selected because of their widespread use in resistance training protocols. This study used a randomized, crossover, repeated-measures design, comprising two separate sessions (separated by at least 72 h). Each subject performed an initial session and experimental session. During the initial session, the instructions of Borg’s CR-10 scale were performed to participants. We then obtained descriptive information from each subject, followed by an anchoring procedure, which determined the range of perceived exertion during the experimental session. During experimental session, three experimental conditions comprising 30% (L), 60% (M), and 90% (H) volume were performed in random order. Volume for each condition was determined by multiplying relative intensity × repetitions (%1RM × number of repetitions). The RPE score, sEMG signal, and velocity were recorded throughout the experimental conditions.

### Participants

The sample size was calculated using the analysis of variance (ANOVA) model with fixed effects, main effects, and interaction analyses. The statistical input parameters were determined with an effect size of 0.4 and an alpha of 0.05. The power was determined to be 0.95 (G*Power 3.1, Bonn University, Germany) [27, 28]; thus, a minimum of 14 participants was indicated for this study. Accordingly, 15 collegiate team sports athletes with no neuromuscular disorders or skeletal muscle injuries who were not taking any medication participated. The participants regularly engaged in national-level collegiate ice hockey competitions, and they all had resistance exercise experience. The descriptive data on the participants were as follows: age (years) 19.40 ± 1.25 (95% confidence interval (CI) [18.77, 20.03]); body mass (kg) 69.59 ± 7.15 (95% CI [65.97, 73.21]); height (cm) 171.80 ± 4.74 (95% CI [169.40, 174.20]); and fat (%) 12.94 ± 4.34 (95% CI [10.74, 15.14]). The 1RM of BS was 134.00 ± 13.69 kg (95% CI [127.07, 140.93]), and 65% of 1RM was 87.10 ± 8.90 kg (95% CI [82.60, 91.60]). The participants were informed about the study’s experimental protocols, measurement items, potential risks, possible discomfort, and benefits, then those who wanted to participate provided written consent. Based on the daily routine and training experience of the participants, they were required to refrain from any resistance exercises 24 h before each testing session. The study was developed in accordance with the Declaration of Helsinki’s ethical guidelines, and the Waseda University Human Ethics Committee (No. 2020–369) approved the experiment. The study was registered in the Japan Registry of Clinical Trials Platform (No. jRCT1030220283, 20/08/2022).

### Orientation and familiarization session

**Fig. 1:**
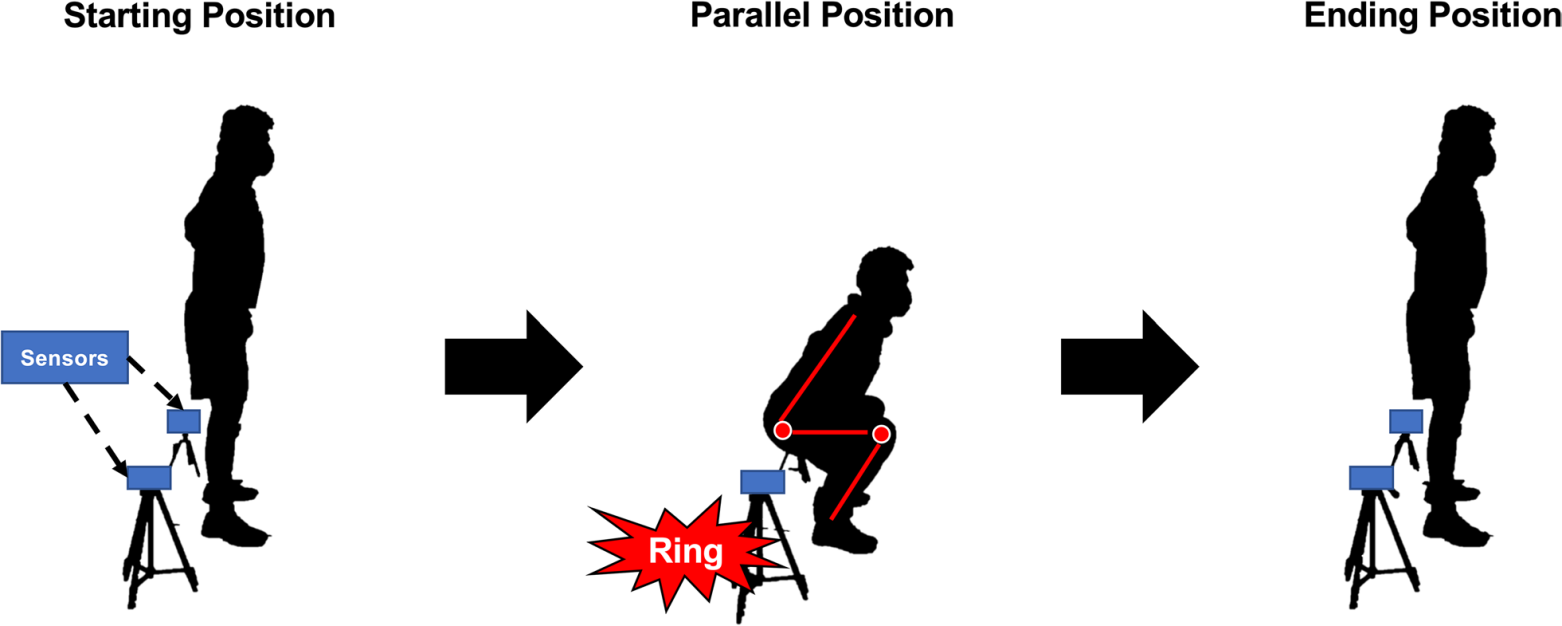
Experimental settings of a successful repetition of a back squat

During the orientation session, the purpose, experimental protocols, measurement items, potential risks, possible discomfort, and benefits were explained to the participants. The experiment was conducted from March to early April. Each subject’s descriptive characteristics were then measured using a bioelectrical impedance device during 12:00 ~ 20:00 in the experimental session (InBody 720 body composition analyzer, Biospace Co. Ltd, South Korea). A line indicating the anatomical landmarks was applied to determine the position of the sEMG electrodes. The distance between the two anatomical landmarks was measured using a tape measure (F10-02, Muratec-KDS Corp., Japan). The location of the electrodes was calculated and determined on the line according to previous recommendations [29]. Subsequently, the 1RM of BS was measured. The BS exercise was performed using a standard Olympic barbell (UESAKA T.E Co., LTD, Japan). When performing BS, the participants were instructed to start from the upright position, then descend in a continuous motion until their thighs were parallel with the floor. A pair of sensors were set at the lowest position of parallel BS to indicate that the participants reached this position during 1RM measurement (TCi Timing System, Brower Timing System LLC, USA) (Fig. 1). When the participants reached this position, the sensors rang immediately. The 1RM determination included the warm-up sets, which were followed by a progressive increase in training load until the participants successfully completed their maximum weight of one repetition. The processes were conducted according to 1RM testing protocols [20].

After the basic measurement, the original Borg’s CR-10 scale was administered with each subject. This scale ranges from 0 (nothing at all) to 10 (extremely strong) and includes standard verbal anchors of perception of effort for intermediate values (in certain previous studies, the terminology was slightly different. For example, “extremely strong” was changed to “very, very strong” on some scales) [21, 30]. A translated version of the scale was adopted and was carefully explained to all participants. The participants were instructed individually. After the instructions, they confirmed that they understood the terminology. After this instruction, the lifting cadence practice was demonstrated to all participants using only a barbell. During the practice, the lifting cadence was determined with a cadence of a 2-s eccentric phase and a 1-s concentric phase, with a 2-s pause phase between repetitions. Raising and lowering cadence were determined based on previous work and recommendations for non-explosive settings [28, 31]. The cadence was controlled by a metronome (one beep per second). After practice, participants performed the anchoring procedure, which determines the range of subjective exertion during experimental trials. The participants were asked to perform a single set of BS exercise until physical failure. The relative intensity of the BS exercise was 65% of 1RM [24]. When performing the BS, the participants were instructed to lower their body to the depth of parallel BS with each repetition until the sensor rings. They also were asked to try their best to follow the metronome. The instructions included the following information: “In the next experimental session, you will be asked to perform a series of BS exercise. During the exercises, we will use this scale to assess your subjective exertion during exercise. The perception of physical exertion is defined as the subjective intensity of effort, strain, discomfort, and fatigue that you feel during exercise. This scale comprises verbal anchors and numbers. Numbers from 0 to 10 represent the range of your subjective feelings, from ‘nothing at all’ to ‘extremely strong.’ The verbal descriptors next to the numbers will help you describe your subjective feelings. The numbers should represent your feelings in the limb you have just lifted. For example, if you are asked to do BS, consider only the feeling in your lower body when reporting your subjective exertion. When reporting your subjective exertion value, please select the nearest number that corresponds to your feeling. After the practice, you will undergo a single set of BS to establish the range of exertion during the experimental session. The perception of exertion when you are sitting down in a relaxed state before any physical activity is equivalent to a score of 0, which means ‘nothing at all’ [32]. We will ask you to perform a single set of BS until physical failure, which means you cannot lift another repetition. When you reach failure, the perceptions of exertion are equivalent to a score of 10, which represents feeling ‘extremely strong’ [18]. You need to remember this range of feelings, and we will ask you to report your score of perceived exertion during later trials based on the range. You need to maintain the cadence of 1 s of the raising phase, 2 s of the lowering phase, and 2 s of pause between repetitions as closely as possible.”

### Experimental session

Before beginning the experimental trial, a series of warm-up exercises was demonstrated to all the participants. The specific warm-up lifts comprised three sets of BS with six repetitions per set at 50% of 1RM [13]. When performing the BS, the sensors also were set at the lowest position of parallel BS. The participants were instructed to lower their body to the depth of parallel BS until the sensor rings throughout the experimental session. After the warm-up lifts, the participants performed an RPE reporting practice using only a barbell. The RPE scale was placed in front of participants, where it could be viewed readily. The lifting cadence comprised a two-second eccentric phase and a one-second concentric phase, with a two-second pause between repetitions controlled by a metronome. The participants were asked to report their feelings of exertion by choosing a number from the CR-10 scale repetition-by-repetition during the two-second pause between repetitions. Participants were asked to consider RPE only based on the range of subjective feelings they established in the anchoring trial.

After the warm-up lifts and RPE reporting practice, the experimental condition trials were implemented randomly. The randomization of the experimental condition trials was performed with Microsoft Excel and blinded to the participants (Microsoft Office 365, Microsoft Corporation, USA). To examine the validity of using RPE as an acute muscle fatigue indicator, all experimental conditions were calculated and implemented based on the results of the anchoring trials. The participants were not informed of the required repetitions beforehand, but they were informed about the penultimate repetition and told to stop immediately after the final repetition. The participants were asked to report their feelings of exertion by choosing a number from the CR-10 scale repetition-by-repetition during the pause between repetitions. Participants were asked to try their best to follow the metronome. After each condition, overall RPE also was obtained from the participants, which represented the total perceived exertion of the latest experimental condition. The following instructions were given to participants before the trial: “You will undergo several sets of parallel BS exercise. You will not be told the required repetitions before the trial until you have reached that number. We will inform you at the second to last repetition. After that, you can stop the exercise after you finish the last repetition. During the exercise, you need to maintain a cadence of 1 s of the raising phase, 2 s of the lowering phase, and 2 s of pause between repetitions as closely as possible. During the pause, you need to report the exertion score of the latest repetition by using a number from the CR-10 scale. After each set, we will ask you to report your overall exertion of the latest set. This exertion score should be considered based on the range of subjective feelings you establish in the anchoring procedure and should be as accurate as possible. When reporting the score, make sure to only consider the exercising limb.” A five-minute rest interval was allowed between conditions. Before the beginning of the next trial, the participants were asked about their RPE to ensure that their subjective exertion had returned to 0. If not, the participants were allowed a longer rest. Overall, all participants reported a 0 on the CR-10 scale at the end of the 5-min rest interval.

### Surface electromyography

The sEMG signals for the vastus medialis, vastus lateralis, and gluteus maximus muscles were obtained using bipolar surface electrodes (ADMEDEC Co., Ltd, Japan). Two Ag/AgCl electrodes were used for each muscle, and the inter-electrode distance was 1 cm. The skin was prepared for the placement of the surface electrodes by shaving, abrasing with sandpaper, then cleansing using alcohol swabs [33]. The electrodes’ location was determined based on recommendations by Barbero et al. [29] to avoid the innervation zone. The signals were recorded using an active differential preamplifier configuration, then transferred to a telemetry device (MARQ MQ-8, Kissei-Com Tech, Japan). The sampling frequency was 1,000 Hz. The signals were processed using an analog digital converter, amplified, then transferred to a computer. Furthermore, a camera (FMVU-03MTC-CS, FLIR Systems, Inc., Canada) was connected to the computer, and the sEMG signals were synchronized with the motion during exercises. The raw signal then was divided into a single repetition and exported for subsequent analysis.

A fourth-order Butterworth band-pass filter (20–450 Hz) was designed to filter noise [26]. This filtered signal then was used to calculate muscle fatigue. sEMG spectral characteristics are affected strongly during dynamic contraction, as discussed in the Introduction section. To address this problem, a new highly sensitive spectral fatigue index (SFI) was adopted to assess muscle fatigue level [11]. SFI ﻿provides a reliable evaluation of muscle fatigue during dynamic contractions compare with traditional sEMG-based fatigue parameters. A fast Fourier transform was applied to calculate the power density spectrum. Spectral moments were used to extract the characteristic features of the power spectral density function and were calculated using the following formula:

1 $$M_{k} = \mathop \smallint \limits_{{f_{\min } }}^{{f_{\max } }} f^{k} \cdot PS\left( f \right) \cdot df$$

in which Mk is a spectral moment of order *k*, PS(f) denotes the power frequency spectrum as a function of frequency *f*, and *f*_*min*_ and *f*_*max*_ delineate the signal’s bandwidth. SFI was calculated as the ratio between orders -1 and 5 based on the following formular:

2 $$SFI = \frac{{\mathop \smallint \nolimits_{{f_{\min } }}^{{f_{\max } }} f^{ - 1} \cdot PS\left( f \right) \cdot df}}{{\mathop \smallint \nolimits_{{f_{\min } }}^{{f_{\max } }} f^{5} \cdot PS\left( f \right) \cdot df}}$$

SFI was calculated for each repetition, and the relative changes in values for each repetition were calculated against the first repetition of the corresponding set. The results from these muscles were averaged to obtain a single variable, which then was used in the statistical analyses. This process was performed using MATLAB R2020a (Mathworks, Natick, MA, USA).

### Velocity loss calculation

In previous studies that focused on the relationship between velocity loss and muscle fatigue assessment, participants were mostly required to perform resistance exercises explosively or under the maximal intended velocity [2, 18]. In order to assess the validity of velocity loss as a muscle fatigue indicator under different resistance exercise settings, the lifting tempo was controlled in this study. The velocity during exercise was recorded using a linear encoder (Fitro Dyne, FITRONiC s.r.o., Bratislava, Slovakia), which was placed directly under the barbell and attached to it with a cable. The sampling frequency was 100 Hz, and upward/downward displacement changes over time during the lifting were recorded and transferred to the computer. The concentric phase’s mean value then was calculated and used in fatigue analyses. Mechanical fatigue was calculated as the percentage loss in velocity from the fastest to the slowest repetition of each condition [2].

### Statistical analyses

The overall RPE, average velocity loss, and average SFI were analyzed for normality using the Shapiro–Wilk test. The average velocity loss of the H condition was not normally distributed. For overall RPE and average SFI, the one-way ANOVA with Bonferroni post hoc testing was used to test for differences between conditions. For average velocity loss, a Kruskal–Wallis H test was used to test for differences between conditions. When analyzing RPE, SFI, and velocity loss during the experimental conditions, 3 × 3 (conditions × repetitions) repeated ANOVA was used to test for main and interaction effects of conditions and times. Furthermore, the Bonferroni post hoc test was used to determine the significant main effects (conditions and repetitions) and interactions between the variables. As each participant performed different repetition numbers in each condition, the first, mid-point, and last repetitions were used for two-way ANOVA.

The relationship between RPE, velocity loss, and SFI during exercise was examined using Spearman’s correlation analysis. The non-overlapping part of the three conditions was used in the correlation analysis [28, 34]. Statistical significance was acceptable at *p* < 0.05 in all analyses. The statistical analysis was performed using SPSS, Version 28.0 (SPSS Inc., USA).

## Results

**Fig. 2:**
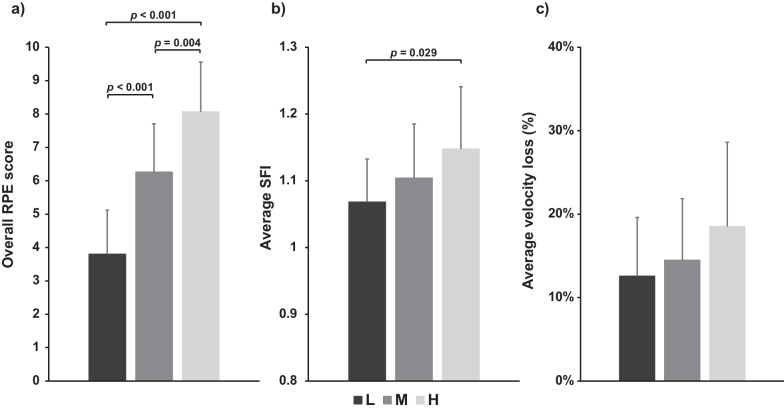
Overall ratings of perceived exertion (RPE) (**a**), average spectral fatigue index (SFI) (**b**), and average velocity loss (**c**) of exercises. The *p* value represents the differences and significant level between experimental conditions

Significant differences in overall RPE (*p* < 0.001, *F* = 34.302, *η*^*2*^ = 0.620) were observed between the L (95% CI: 3.062–4.538), M (95% CI: 5.529–7.005), and H (95% CI: 7.329–8.805) conditions (Fig. 2a). Significant differences also were observed in average SFI between the L and H conditions (*p* = 0.034, *F* = 3.675, *η*^*2*^ = 0.149, 95% CI of L: 1.026–1.110; 95% CI of M: 1.062–1.146; 95% CI of H: 1.106–1.189) (Fig. 2b). Regarding average velocity loss, no significant difference was observed between both conditions (*p* = 0.172, 95% CI of L: 0.085–0.167; 95% CI of M: 0.102–0.187; 95% CI of H: 0.127–0.244) (Fig. 2c).

**Fig. 3:**
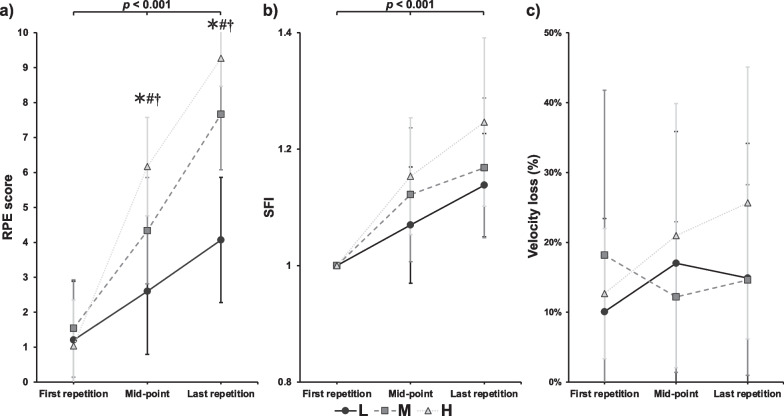
Rating of perceived exertion (RPE) (**a**), spectral fatigue index (SFI) (**b**), and velocity loss (**c**) during low (L, circle with solid lines), medium (M, square with dashed lines), and high (H, triangle with dotted lines) volume condition of exercises. *represents a significant difference as L compared with other conditions, *p* < 0.001; #represents a significant difference as M compared with other conditions, *p* < 0.001; †represents a significant difference as H compared with other conditions, *p* < 0.001; The *p* value indicates the overall main effects and significant level for repetitions

The significant overall main effect for repetitions was observed in the RPE (*p* < 0.001, *F* = 261.286, *η*^*2*^ = 0.949) and SFI (*p* < 0.001, *F* = 84.955, partial *η*^*2*^ = 0.859) throughout the BS trials. As for velocity loss, no significant effect on repetitions was observed (*p* = 0.871, *F* = 0.139, partial *η*^*2*^ = 0.011). Significant differences were observed in RPE between conditions at the mid-point (*p* < 0.001, *F* = 61.475, partial *η*^*2*^ = 0.815, 95% CI of L: 1.601–3.599; 95% CI of M: 3.492–5.157; 95% CI of H: 5.386–6.947) and last repetition (*p* < 0.001, *F* = 81.547, partial *η*^*2*^ = 0.853, 95% CI of L: 3.075–5.059; 95% CI of M: 6.787–8.546; 95% CI of H: 8.824–9.709) (Fig. 3a). When comparing SFI, no significant effect for condition or interaction was observed (condition: *p* = 0.059, *F* = 3.143, partial *η*^*2*^ = 0.183; interaction: *p* = 0.076, *F* = 2.244, partial *η*^*2*^ = 0.138) (Fig. 3b). Significant effects for conditions were observed in velocity loss (*p* = 0.026, *F* = 4.242, partial *η*^*2*^ = 0.261), but pairwise comparisons indicated no significant difference between conditions (Fig. 3c).

**Fig. 4:**
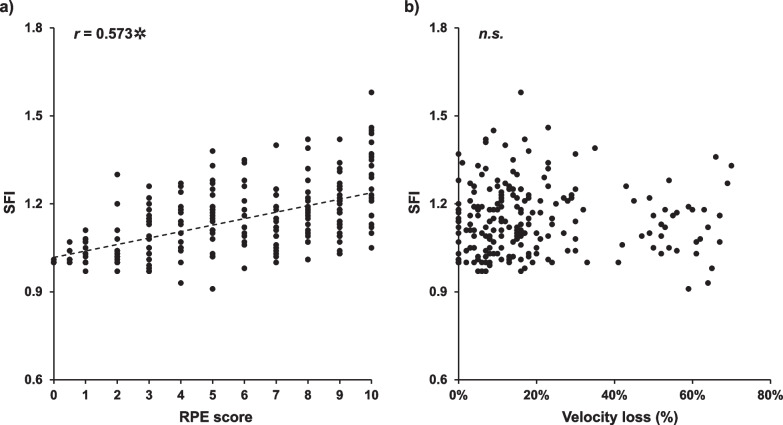
Spearman’s Rho between spectral fatigue index (SFI), ratings of perceived exertion (RPE) scores (**a**), and velocity loss (**b**) during the exercises. *represents the significant level of correlation coefficients, *p* < 0.001

Overall, 215 BS repetitions were used in the correlation analysis. The Spearman correlation analysis results are shown in Fig. 4. A significant relationship was found between RPE and SFI (*r* = 0.573, *p* < 0.001) (Fig. 4a); however, no significant relationship was observed between RPE and velocity loss during exercise (Fig. 4b).

## Discussion

This study’s purpose was to investigate the validity of using RPE to assess exercise-induced muscle fatigue and to compare this with the use of velocity loss as a fatigue indicator during BS exercise. This study’s most important contributions were as follows: 1) RPE and SFI changed correspondingly, indicating that muscle fatigue exhibits similar increases in perceptual responses, and 2) a significant RPE-SFI correlation was observed, indicating that the RPE could be used as a muscle fatigue indicator when performing BS exercise. However, 3) velocity loss did not reflect muscle fatigue correctly when the exercise target is no longer an explosive performance.

Subjective exertion raises in response to proximal and distal changes such as motor unit recruitment and glycolysis when performing resistance exercises with dynamic contractions [14, 35–37]. For example, Lagally et al. observed that muscle activation level and RPE changed correspondingly, indicating RPE is sensitive to relative intensity changes of resistance exercise [14]. In present study, the relative intensity was constant between conditions to eliminate the possibility that RPE was dependent on relative intensity. Therefore, the reason of significant difference in RPE can be attributed to the changes in duration of consecutive lifting phase. Some previous studies indicated that more repetitions could induce an impact on glycolysis and RPE responses [1, 13]. In agree with these previous findings, with increasing volume, more repetitions were performed continuously, which have induced more severe intramuscular perturbation (e.g., hydrogen ion concentration in skeletal muscles). These muscle contraction-related physiological changes can affect afferent feedback from the terminal end of myelinated and unmyelinated nerve fibers in skeletal muscle, and the perception of exertion would increase [36–38]. Consequently, RPE increased statistically significant in present study. Moreover, the dramatic disruption in homeostasis has been found to affect muscle fiber conduction velocity, indicated by power spectrum shifts [7, 39]. In our study, SFI entailed the responses of changes in power spectral characteristics, which very likely were associated with fatigue-induced metabolic byproduct accumulation [40]. As a result, SFI increased significantly during exercise. Accordingly, these corresponding changes suggest that RPE reflects muscle fatigue responses to BS exercise.

In the present study, we assessed the relationship between RPE and SFI using data collected repetition-by-repetition, with a significant correlation observed between them. Furthermore, a significant relationship was observed between the RPE and SFI. These results indicated that RPE could be used as an exercise fatigue predictor, which makes sense because RPE integrates more physiological-related information induced by resistance exercises [18, 34]. Conventionally, RPE is measured upon completion of required sets and/or sessions, as represents of subjective feelings of discomfort overall [25, 41]. These results provided a new method of monitoring resistance exercises using RPE. For example, coaches and personal trainers could place the RPE scale where participants can see them and obtain perceptual responses at pre-determined repetitions (e.g., first, median, and last repetition) or repetition-by-repetition. By using this method, they can grasp fatigue conditions within the process of repetition numbers and, thus, reduce injury risks induced by acute muscle fatigue [42]. These results also correspond with previous findings and have expended the use of RPE-based muscle fatigue assessment from single-joint to lower-body multi-joint resistance exercises [28]. Accordingly, the results suggest that RPE could be used as a muscle fatigue-predicting tool when performing BS exercise.

As for the results on velocity loss, an overall main effect of repetitions was not found, indicating that velocity loss failed to increase corresponding to the development of muscle fatigue. Furthermore, the significant relationship between muscle fatigue and velocity loss was not observed in all analyses. It could be concluded that the experimental setting affected the precision of velocity loss as a measure of fatigue. In previous research that focused on the relationship between velocity loss and fatigue-related measures, participants were required to perform the exercise under their maximal intended velocity, and some studies were performed until physical failure [2, 16, 18]. However, as we discussed in the Introduction, in the contexts of muscular hypertrophy, rehabilitation, and lack of exercise experience, it is inappropriate for people to perform the exercises explosively and/or until physical failure. Accordingly, we used a cadence-controlled design to assess the validity of velocity loss as a fatigue indicator in various resistance exercise situations. Under these experimental settings, the validity of velocity loss seemed to offer very limited precision when measuring fatigue mechanically. Although a significant main effect on condition was observed (*p* = 0.026), it could be concluded that velocity loss only increases significantly when the resistance exercise gets very close to volitional failure (e.g., 90% of the volume until failure). Furthermore, using velocity loss as a muscle fatigue indicator is questionable if participants are not required or cannot perform the exercises explosively. Accordingly, we can conclude that RPE reflects muscle fatigue responses more correctly than velocity loss when BS exercise are no longer performed explosively and/or when the muscle gets very close to volitional failure. Furthermore, caution should be employed when using velocity loss as a muscle fatigue indicator in some resistance exercise situations (e.g., hypertrophy, beginner, and rehabilitation programs).

An unexpected finding was that significant differences between conditions did not occur in SFI during the exercise (Fig. 3b). At first, the small sample size was blamed. Although the significant difference between conditions in during-exercise SFI was not observed, the *p* value for conditions is 0.059, and for interaction, it is 0.076, which is very close to statistical significance. Similar changes in SFI would be more likely with a larger sample size. Another reason may be differences in muscle fiber composition and metabolic responses in leg muscles [43–45]. For example, some studies have found that Type-I fiber distributions in the vastus lateralis are larger than those in arm muscles [44, 45]. These slow-twitch fiber compositions in legs have relatively lower conduction velocity, which may affect sEMG spectral characteristics [46]. This corresponds with our previous finding, which indicated a more sophisticated muscle fatigue response in single-joint resistance exercise in legs [28]. Thus, sample size and leg muscles’ physiological characteristics might have affected SFI results during exercises.

Present study has some limitations. First, only the original Borg’s 10-grade scale was examined in the present study. Different results might be observed if different RPE scales were adopted (e.g., OMNI-RES scale). Second, other physiological responses such as metabolic and endocrine variables were not assessed in this study. Some researchers have indicated that different lifted volume might transmit into different neuroendocrine and/or metabolic responses. These perturbations would potentially affect perceived exertion [13]. Third, although we specifically excluded contributions to the RPE scale that were not related to the BS trials, it would still be difficult for participants to separate the subjective exertion induced by other factors (e.g., breathlessness) during intensive lifting tasks [47]. Last, as all the participants were male, our result seems to lack insight on sex-specific responses. For example, Otto et al. found that gender differences affect muscle fatigue strategies of some certain muscles [26]. Thus, future studies should examine different RPE scales, other physiological-related responses (e.g., metabolic and endocrine measures), and sex-specific characteristics of RPE and muscle fatigue quantifications during resistance exercises.

## Conclusion

The present study demonstrated that SFI and RPE changed correspondingly, indicating a link between perceptual responses and muscle fatigue. We concluded that muscle fatigue exhibits similar increases in perceptual responses when BS exercise are performed. Furthermore, we observed a significant relationship between SFI and RPE, indicating that RPE scores could be used as a muscle fatigue indicator ﻿in clinical and sports situations. These results demonstrate that RPE could be used as a predictor for assessing muscle fatigue during BS exercise. For example, physical therapists and coaches can use RPE assessments during BS exercise as a timely and reliable indicator of muscle fatigue. However, velocity loss failed to exhibit similar effects on muscle fatigue, with no significant relationship found between velocity loss and SFI. Velocity loss is inappropriate for assessing fatigue when the exercise target is not an explosive performance and/or performed until physical failure. Furthermore, practitioners should not use velocity loss as a muscle fatigue indicator in some resistance exercise situations, such as hypertrophy, beginner, and rehabilitation programs.

## Data Availability

All data generated or analyzed during this study are included in article. Code of Matlab generated during the electromyography analyses are available from the corresponding author by request.

## Abbreviations

ANOVA: Analysis of variance
BS: Back squat
H: High volume condition
L: Low volume condition
M: Medium volume condition
RM: Repetition maximum
RPE: Ratings of perceived exertion
sEMG: Surface electromyography
SFI: Spectral fatigue indices
